# From common to rare: repurposing of bempedoic acid for the treatment of glycogen storage disease type 1

**DOI:** 10.1186/s12263-023-00733-2

**Published:** 2023-09-18

**Authors:** Anibh M. Das

**Affiliations:** Department of Paediatrics, Clinic for Paediatric Kidney, Liver and Metabolic Diseases, Paediatric Metabolic Medicine, Carl Neuberg Str. 1, 30625 Hannover, Germany

**Keywords:** Bempedoic acid, Glycogen storage diseases type 1, Ketone bodies, Malonyl-CoA

## Abstract

Hypoketotic hypoglycaemia is a biochemical hallmark of glycogen storage disease type 1 (GSD1). This is due to inhibition of carnitine-palmitoyl transferase 1 by malonyl-CoA. This inhibits the influx of long-chain fatty acids into the mitochondrial matrix for fatty acid oxidation. This leads to reduced hepatic ketogenesis and impaired energy production in the liver and kidney. Hypoketotic hypoglycaemia may result in CNS symptoms due to energy depletion.

Recently, it was reported that enzymes involved in mitochondrial long-chain fatty acid oxidation are upregulated in PBMC from patients suffering from GSD1.

I suggest that administration of the prodrug bempedoic acid results in reduced production of malonyl-CoA by inhibiting the ATP-citrate lyase, thus releasing the block of mitochondrial long-chain fatty acid influx. These fatty acids could make use of the increased capacity of fatty acid oxidation as observed in PBMC recently. In the liver, ketogenesis is activated, and energy production is increased in both the liver and kidney. This could result in improved metabolic control and avoidance of cerebral energy depletion.

Bempedoic acid is approved as medication in adult patients with hypercholesterolaemia and mixed dyslipidaemia. Repurposing bempedoic acid for the use in GSD1 may improve metabolic control in GSD1.

In a recent research paper published in this journal, Rossi and coworkers report on mitochondrial reprogramming in peripheral blood mononuclear cells (PBMC) of patients with glycogen storage disease type 1a [1].

Glycogen storage disease type 1 (GSD1, von Gierke disease) is an ultrarare disorder with a prevalence of 1:100,000; it is inherited in an autosomal-recessive manner. Two subtypes are known: GSD1a is due to deficiency of glucose 6-phosphatase in the endoplasmic reticulum (ER), while in GSD1b, the import of glucose 6-phosphate into the ER is disturbed. Typical clinical findings are hepato- and nephromegaly, short stature and in GSD1b additionally frequent bacterial infections due to neutropenia as well as inflammatory bowel disease. Characteristic laboratory findings are hypoketotic hypoglycaemia, hyperlipidaemia, hyperuricaemia and elevated lactate and alanine levels. These findings result from the accumulation of glucose 6-phosphate [2].

The lack of ketogenesis during hypoglycaemia may result in seizures and coma due to cerebral energy depletion. Frequent carbohydrate-rich feedings and uncooked cornstarch can prevent hypoglycaemic episodes and have improved the long-term outcome; however, the diet is demanding and has considerable unmet needs [3].

The biochemical basis for reduced ketogenesis in GSD1 is the accumulation of malonyl-CoA from glucose 6-phosphate. Malonyl-CoA blocks the transport of long-chain fatty acids (LC-FA) into the mitochondrial matrix via the carnitine-acylcarnitine shuttle by inhibiting the coupling of LC-FA to carnitine catalysed by carnitine-palmitoyl transferase 1 (CPT1) (Fig. 1) [4]. In the liver, LC-FA undergo mitochondrial fatty acid oxidation which is essential for ketogenesis as well as hepatic energy production. We have previously shown in patients with GSD1 that an MCT-enriched diet can partially bypass the blocked carnitine-acylcarnitine shuttle as medium chain-fatty acids can enter the mitochondrial matrix without the carnitine-acylcarnitine shuttle. Thus, metabolic control can be improved in GSD1 patients [5].

**Fig. 1:**
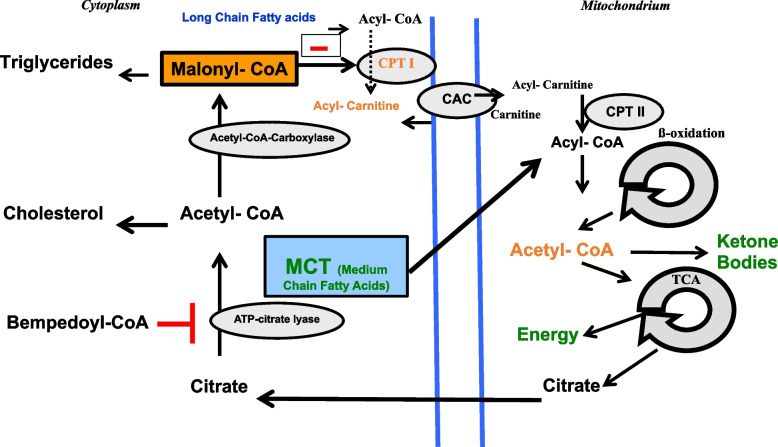
Bempedoic acid and GSD1. Malonyl-CoA, which is synthesized from cytosolic acetyl-CoA, blocks the entry of long-chain fatty acids into the mitochondrial matrix by inhibition of CPT1. Bempedoyl-CoA inhibits ATP-citrate lyase. CAC, carnitine-acyl-carnitine exchanger; CPT, carnitine palmitoyltransferase; TCA, tricarboxylic acid cycle

Interestingly, Rossi and coworkers found upregulation of various mitochondrial enzymes in PBMC, including CPT1, which play a role in fatty acid oxidation. Thus, the theoretical capacity of fatty acid oxidation was increased in PBMC of GSD1a patients. PBMC are assumed to reflect metabolic changes in other organs and can be used as surrogate cell type for other metabolically more active and relevant cells like hepatocytes and kidney cells [6]. Therefore, the fatty acid oxidation capacity is presumably increased in hepatocytes from GSD1 patients. Nevertheless, the flux of fatty acid oxidation is functionally inhibited by cytosolic malonyl-CoA accumulation. It seems tempting to release the malonyl-CoA-induced block in an attempt to increase energy production as well as ketogenesis via long-chain fatty acid oxidation, thus improving metabolic control.

In 2020, bempedoic acid was approved by FDA and EMA for the treatment of hypercholesterolaemia and mixed dyslipidaemia. This is the most common inherited metabolic disorder with a prevalence of 1:200–250.

Bempedoic acid is a prodrug which has to be activated to bempedoyl-CoA catalysed by very long-chain acyl-CoA synthase 1 (ACSVL1). This enzyme is present in the liver and to a lesser extent in kidney [7, 8]. Therefore, substantial concentrations of the active drug can only be found in the liver and kidney.

Bempedoyl-CoA inhibits the ATP-citrate lyase (ACL) that cleaves cytosolic citrate from the mitochondrial citric acid cycle to acetyl-CoA and oxaloacetate, thus inhibiting the de novo synthesis of cholesterol and triglycerides as well as malonyl-CoA production (Fig. 1, left part) [7, 9]. Decreased cytosolic malonyl-CoA leads to a release of the blocked influx of LC-FA into the mitochondrial matrix. Thus, ketone bodies can be formed as alternative cerebral fuel in the liver during hypoglycaemia using the upregulated mitochondrial fatty acid oxidation pathway (Fig. 1). Furthermore, hepatic energy production improves, and synthesis of cholesterol and triglycerides is reduced.

In dyslipidaemias, BA is safe, and mild elevations of uric acid were observed in some patients. Uric acid may rise slightly, as BA glucuronide competes with uric acid for the same renal transporter [7, 8]. In most patients with dyslipidaemia, elevation of uric acid in blood is not critical.

In summary, BA could improve metabolic control, namely the capacity to produce ketone bodies during hypoglycaemic spells, hyperlactataemia and lipid elevation by using the upregulated fatty acid oxidation pathway as recently described [1]. Clinical studies are required to prove the validity of this biochemical concept.

## Data Availability

Not applicable.

## Abbreviations

ACL: ATP-citrate lyase
ACSVL1: Very long-chain acyl-CoA synthase 1
BA: Bempedoic acid
CPT1: Carnitine-palmitoyl transferase 1
ER: Endoplasmatic reticulum
GSD1: Glycogen storage disease type 1
LC-FA: Long-chain fatty acids
PBMC: Peripheral blood mononuclear cells
